# Klippel-Feil Syndrome With Isolated Facial Dysmorphism: A Clinical Conundrum With Resemblance to Adenoid Facies

**DOI:** 10.7759/cureus.58466

**Published:** 2024-04-17

**Authors:** Nimisha Patil, Shraddha Jain, Nikhil Kumar, Rinkle Gemnani

**Affiliations:** 1 Otolaryngology - Head and Neck Surgery, Jawaharlal Nehru Medical College, Datta Meghe Institute of Higher Education and Research, Wardha, IND; 2 Orthodontics and Dentofacial Orthopedics, Kusum Devi Sunderlal Dugar Jain Dental College & Hospital, Kolkata, IND; 3 Medicine, Jawaharlal Nehru Medical College, Datta Meghe Institute of Higher Education and Research, Wardha, IND

**Keywords:** craniofacial dysmorphism, retrognathia, klippel-feil syndrome, crowded dentition, cervical vertebrae fusion

## Abstract

Klippel-Feil syndrome (KFS) is a triad comprising cervical spine fusion, a low posterior hairline, and constrained neck movement. This triad is not universally present. The most frequent accompaniment is Sprengel's scapula deformity. According to the Feil classification, Class 1 (C1) is an immense fusion of many cervical vertebrae, Class 2 (C2) is a fusion of one or two vertebrae only, and Class 3 (C3) is coupled with thoracic and lumbar spinal vertebral fusion in addition to the fusion of the cervical vertebrae. Clarke's categorization of KFS includes other associated anomalies. The different classification systems for KFS have been made by the different specialists to whom patients may present, which include orthopedic surgeons, neurosurgeons, orthodontists, faciomaxillary surgeons, cardiologists, and pediatricians. This anomaly being rare and the lack of universally accepted classification may lead to confusion regarding the identification of the syndrome, especially the Clarke Type 3 with isolated facial dysmorphism may go undiagnosed. We report a case with KFS-Clarke Type 3 with isolated facial dysmorphism and Feil Type 2 with the fusion of C2-C3 cervical vertebrae, detected as an incidental radiologic finding, and initial impression of adenoid facies. Hence, this case also highlights the contrasting features between the facial dysmorphism of Clarke Type 3 KFS and adenoid facies.

## Introduction

Klippel-Feil syndrome (KFS) is defined as a rare, congenital skeletal deformity that is unusual, having an incidence of one in 40,000-42,000 births [1]. It is caused by failure of normal segmentation of any two of the seven cervical vertebrae and leading to fusion. The fusion in the lower spine is not considered as KFS. KFS is marked by cervical vertebral fusion, hence leading to limited neck mobility, and it may be encountered in association with multiple skeletal deformities, oro-maxillofacial deformities such as cleft palate, Sprengel deformity, spina bifida, airway obstruction leading to respiratory difficulties, and cardiac malformations [2].

KFS was originally classified by Maurice Klippel and Andre Feil who divided this syndrome into three types depending on the number and position of the vertebral fusion. Type 1 includes a massive fusion of vertebrae; Type 2 includes one or two vertebrae; and Type 3 is a combination of Types 1 and 2 with thoracic and lumbar vertebral fusion [1]. As this classification does not include other associated syndromes; hence, there was a need for other classification systems, which would encompass other anomalies to identify syndromic associations to foster holistic patient management. One such classification was given by Clarke [3].

The dentofacial features of KFS are less known to otolaryngologists. The more common condition with dentofacial anomalies known to them is adenoid facies. Here, we present a rare case of KFS without any associated syndromes, associated with retrognathia with dentofacial features, Clarke Type 3, initially misdiagnosed for adenoid hypertrophy, and diagnosed as an incidental radiologic finding. Moreover, we discuss the comparative dentofacial features of the two conditions.

## Case presentation

**Figure 1:**
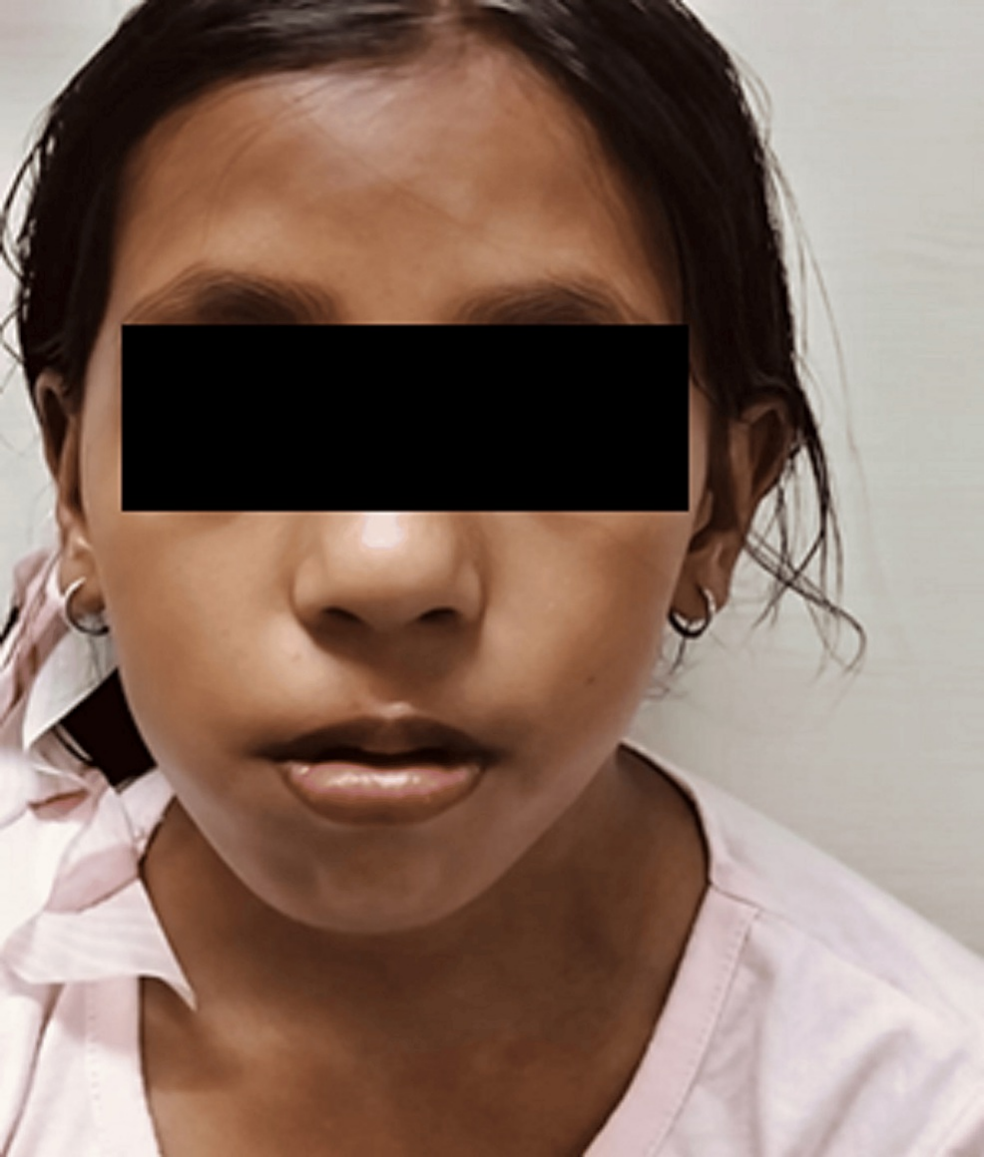
Facial dysmorphism in the patient

**Figure 2:**
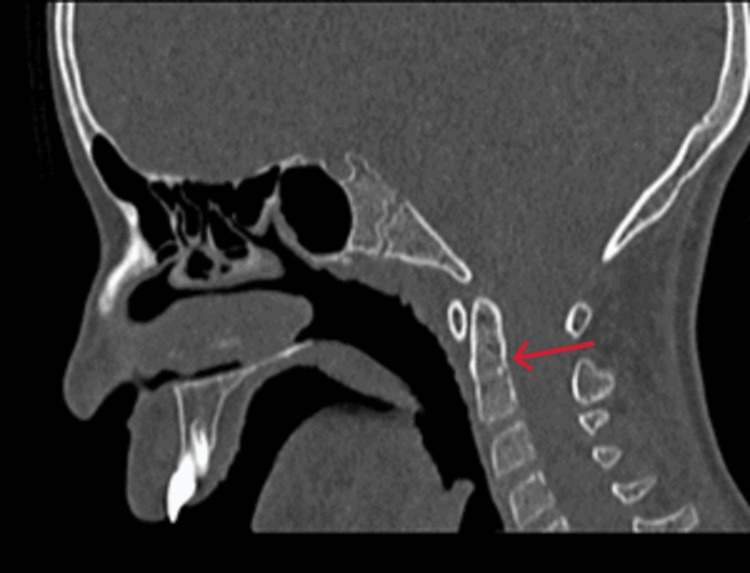
CT of the paranasal sinus sagittal view showing the fusion of C2–C3 cervical vertebrae (marked by a red arrow)

A nine-year-old female patient reported to the otorhinolaryngology department with complaints of mouth breathing since childhood. The patient had complaints of disarticulated speech. On external examination, the facial analysis revealed elongated facies, acute-angled nasolabial folds, open-mouth breathing, and retrognathia (Figure 1). Dentofacial deformities comprised of facial asymmetry with deviation to the left side, with an anterior open bite and downward tongue position, crowded dentition, and high arched palate with retrognathia. Based on the history, an initial diagnosis of adenoid facies was made; hence, further radiological investigations were conducted. On the evaluation of cervical vertebral radiographs, an incidental finding of fusion of the body of the C2 and C3 vertebra was revealed. To confirm the finding, a CT study of paranasal sinus was conducted, which reported the fusion of the body of C2 and C3 vertebrae (Figure 2), suggestive of Type 2 KFS as per Feil classification based on extent of vertebral fusion [2], Class 3 as per the classification given by Clarke addressing genotypic and phenotypic heterogenicity because of the isolated facial dysmorphism, and Type 1 as per the classification based on prognostic value laid by Samartzis et al. [4].

The patient was referred to the orthodontic and orthognathic departments for restorative and orthodontic treatment, but because of financial constraints, the patient was not willing to receive further management.

## Discussion

KFS is an unusual skeletal congenital deformity characterized by a union of two or more cervical vertebrae that may coexist with other congenital disorders. The classic triad of short neck, low-lying posterior hairline, and limited range of neck mobility is observed in KFS as described by Samartzis et al. [4]. However, in another series by Pizzutillo et al. [5], only one or more of the components of the triad may be present, with a maximum percentage (76%) of patients having a limited cervical range of motion.

Our observations were found to be similar in that fused cervical segments were detected as incidental findings on radiologic investigations. In our case, there was evidence of C2-C3 fusion with a limited range of neck motion without a triad of KFS and any syndromic associations. Because of the many similarities in facial and oro-maxillary appearances, it is very common to miss the diagnosis, and because of unusual presentation, it can be misdiagnosed as adenoid facies. This conundrum was faced by us in our case where the patient, because of a history of mouth breathing and snoring, was evaluated and on examination for a high arch palate and crowded dentition, pointing the diagnosis toward adenoid hypertrophy. On further evaluation, there was no adenoid hypertrophy, and an incidental finding of fusion of cervical vertebrae was found, which changed the diagnosis to KFS.

KFS also shows an association with cardiac anomalies ruled out in this case by 2D Echocardiography and cardiac assessment. Patients with this condition are advised to have regular intravenous urograms, ultrasonography, and urine investigations to eliminate renal anomalies [6]. In the present case, no such abnormality was detected on the ultrasound abdomen and pelvis, which made this case an isolated KFS without any association with other syndromes, making this a unique case that could have been underdiagnosed. Various cardiac and renal manifestations are associated with KFS, which changes the type as per the various classifications and, hence, the management [7].

KFS is usually diagnosed at later ages; hence, there is a chance of missing out on the diagnosis as it is also associated with various other congenital syndromes like the Pierre Robin sequence [8]. The former consists of both prognathia and retrognathia whereas Pierre Robin sequence consists of micrognathia, posterior placement of the tongue, and airway obstruction and hence can be misinterpreted. KFS can also be associated with Treacher Collins syndrome, which consists of facial dysmorphism of pharyngeal arches causing malformation of dentofacial features and ultimately leading to feeding difficulty [9]. KFS is distinguished from Marfan syndrome, where the latter presents with a fusion of the atlantooccipital joint and may have a short neck with restricted neck movements and, hence, needs to be distinguished in radiological studies [10]. There is a possibility of missing the diagnosis because of the absence of other associated syndromes and can be misdiagnosed as the presentations of facial dysmorphism may be confused with the other syndromic facies. The features we encountered in this case, which were common and differentiating both KFS and adenoid hypertrophy, helped us to come to the diagnosis (Table 1).

**Table 1:** Dentofacial anomalies associated with Klippel–Feil syndrome versus adenoid hypertrophy [11]

Sr No	Features	Klippel-Feil Syndrome	Adenoid Hypertrophy
1	Retrognathia	Present [4]	Absent
2	High arched palate	Present [4]	Present [11]
3	Mouth breathing	Present [4]	Present [11]
4	Dentition-Maloclussion	Class III [4]	Present [11]
5	Elongated facies	Present	Present [11]
6	Cleft palate	Present [12]	Absent
7	Bifid uvula	Present [12]	Absent

In this case, the patient was counseled for orthognathic treatment and was referred to the concerned department for further management. As KFS is associated with multiple organ involvement other than the head and neck region, there is always a risk of serious complications, which makes having an integrated management plan necessary in such cases, which may include surgical specialists such as neurosurgeons, otolaryngologists, orthopedists, pediatricians, and oro-maxillofacial surgeons.

## Conclusions

The present case of a female with KFS associated with isolated facial dysmorphism, Type 2 as per Feil, Class 3 as per the classification given by Clarke, and Type 1 as per the classification laid by Samartzis; incidentally diagnosed on radiological investigations, which were done considering it a case of adenoid hypertrophy, highlights the need of a multidisciplinary approach for devising a single unified classification relevant to all specialties. The patient because of malocclusion and dentofacial problems may present to an otorhinolaryngologist for mouth breathing and snoring. They should be aware of this entity and its contrasting features with adenoid facies for the execution of a proper line of management, comprising orthodontic treatment.
